# Computing within-study covariances, data visualization, and missing data solutions for multivariate meta-analysis with metavcov

**DOI:** 10.3389/fpsyg.2023.1185012

**Published:** 2023-06-20

**Authors:** Min Lu

**Affiliations:** Division of Biostatistics, Department of Public Health Sciences, Miller School of Medicine, University of Miami, Miami, FL, United States

**Keywords:** multivariate meta-analysis, effect sizes, variance-covariance matrix, multiple imputation, confidence intervals

## Abstract

Multivariate meta-analysis (MMA) is a powerful statistical technique that can provide more reliable and informative results than traditional univariate meta-analysis, which allows for comparisons across outcomes with increased statistical power. However, implementing appropriate statistical methods for MMA can be challenging due to the requirement of various specific tasks in data preparation. The metavcov package aims for model preparation, data visualization, and missing data solutions to provide tools for different methods that cannot be found in accessible software. It provides sufficient constructs for estimating coefficients from other well-established packages. For model preparation, users can compute both effect sizes of various types and their variance-covariance matrices, including correlation coefficients, standardized mean difference, mean difference, log odds ratio, log risk ratio, and risk difference. The package provides a tool to plot the confidence intervals for the primary studies and the overall estimates. When specific effect sizes are missing, single imputation is available in the model preparation stage; a multiple imputation method is also available for pooling the results in a statistically principled manner from models of users' choice. The package is demonstrated in two real data applications and a simulation study to assess methods for handling missing data.

## 1. Introduction

Multivariate meta-analysis (MMA) is a statistical technique of combining multiple effect sizes, either of the same type or different types, from different studies to produce one overall result. It allows for within-study dependence among effect sizes caused by the fact that multiple outcomes are obtained from the same samples in the primary studies. This dependence could increase the Type I error rate and lead to inaccurate estimates of study effects (Becker, 2000; Nam et al., 2003; Riley, 2009; Jackson et al., 2011). Although there are many R packages available for univariate meta-analysis, resources for MMA are limited in terms of data preparation and visualization (Michael Dewey, 2021). There are available R packages (see Table 1) designed for fitting MMA models, but they assume that the within-study variance-covariance matrices of the effect sizes from all studies are pre-computed by the users. Therefore, these packages may be unattractive in practice. For example, in some MMA application articles, univariate meta-analysis is still adopted even though several effect sizes are extracted from the same study (Sebri et al., 2021; Watters et al., 2021). Conducting statistically principled MMA confronts challenges, which are as follows:

1. It is challenging to compute the covariances among effect sizes for non-statisticians;
2. It lacks data visualization tools;
3. It suffers greatly from the missing data problem.

**Table 1:** R-packages for conducting MMA.

**Package**	**Unique features^†^**
metavcov	Preparing within-study variances and covariances; plotting confidence intervals^*^
mixmeta	Multiple choices for mixed-effect model fitting including maximum likelihood, restricted maximum likelihood, method of moments, and variance components
metaSEM	Meta-Analysis using Structural Equation Modeling; plotting model structures
metafor	rma.mv() accommodates repeatedly measured outcomes^*a*^
mmeta	Fitting Bayesian models for binary outcomes^*b*^
metaCCA	Detecting genetic association with shrinkage for high dimensional outcomes^*c*^
CopulaREMADA	Fitting copula mixed models for diagnostic test accuracy studies^*d*^
xmeta	Testing and visualizing publication bias for bivariate meta-analysis^*e*^

† In general, all the listed R-packages can conduct MMA. This table highlights their unique features, rather than major features. They have many features to explore.

* While this study focuses on demonstrating the utility of metavcov for mixmeta and metaSEM, it can provide similar benefits to other packages as well.

a Viechtbauer (2010);

b Luo et al. (2014);

c Cichonska et al. (2016);

d Nikoloulopoulos (2020);

e Hong et al. (2020).

**Figure 1:**
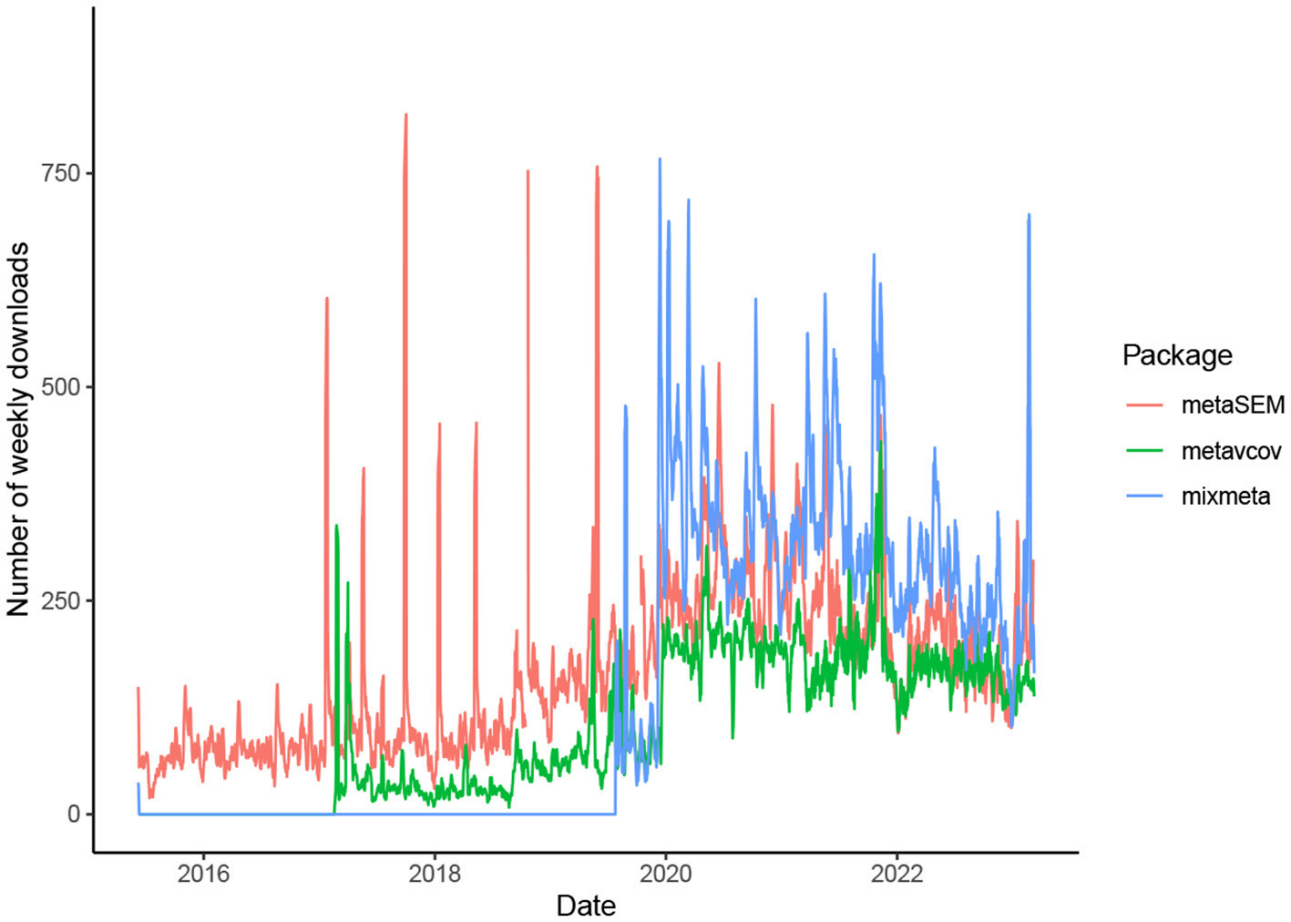
Number of weekly downloads from CRAN for the three R packages useful for conducting MMA. The package metavcov was initially released in 2017, which is designed for preparing variance-covariance matrices of effect sizes for packages metaSEM and mixmeta that were released in 2015 and 2019, respectively (mixmeta is a new version of the package mvmeta which was initially released in 2011).

The availability of generalizable, user-friendly software packages facilitates the incorporation of MMA into various fields of science. The package metavcov aims to provide useful tools for conducting MMA in R (R Core Team, 2016) with examples of how it can provide aid for easy, efficient, and accurate computer programs (Lu, 2017). It is not designed to replace a parameter estimation package for MMA, such as mixmeta and metaSEM (Aloe et al., 2014a,b; Gasparrini, 2019; Cheung, 2021), but to provide additional specialized tools. It was initially released in 2017 for computing variance-covariance matrices of effect sizes and has attracted growing downloads as shown in Figure 1. Its new version addresses all the above three points. For point 1, formulas and references are provided in the next section for computing covariances. Tutorials are given to guide users to use R functions that can accommodate different types of effect sizes and their variance-covariance matrices for preparing desired input arguments for packages mixmeta and metaSEM as examples. Note that since the diagonal elements of the variance-covariance matrix are the variances of the estimated effect sizes, this package can also be used for preparing univariate meta-analysis.

For point 2, the metavcov package introduces a function for confidence interval plots. Although forest plots are used for displaying effect sizes from all studies and their overall estimator in the univariate meta-analysis (Schwarzer, 2007; Boyles et al., 2011; Sedgwick, 2015; Rücker and Schwarzer, 2021), they are inappropriate for MMA because forest plots require a symbol on each confidence interval that is proportional to the weight for each study, but the weighting mechanism in MMA is too complex to be visualized. Therefore, for MMA, the tool for displaying sample effect sizes and their overall estimators is a confidence interval plot without displaying weights. Studies with smaller standard errors for the effect sizes would contribute more to the overall estimators, and these effect sizes have narrower confidence intervals. Hence, although a confidence interval plot does not directly reflect weights for each study, it could provide quite sufficient information for the users.

For point 3, missing data problems in meta-analysis are often tackled through methods of omission, single imputation, such as augmenting the missing values with the sample-size-weighted mean or zero, multiple imputation, or integrating the missing pattern into the estimation method such as Higgins et al. (2008)'s two-stage method or methods employing a Bayesian framework (Rubin, 1976; Sutton et al., 2000; Allison, 2001; Schafer and Graham, 2002; Graham, 2009; Yuan and Little, 2009; Mavridis and Salanti, 2013; Little and Rubin, 2019). Since MMA requires far more statistical records from each study than univariate meta-analysis, it is harder to get a complete list of effect sizes and sample sizes. Missing data are often omitted by default in packages mixmeta and metaSEM. Meanwhile, mixmeta provides the function mixmetaSim to simulate responses that can be potentially used for missing data imputation, and metaSEM supports handling missing covariates using full information maximum likelihood in meta-regression. However, these options do not consider or distinguish different types of effect sizes in detail. For example, when calculating the covariance between two odds ratios, we need to know the sample size *n*_*jkt*_ that counts for individuals reporting both outcomes, *j* and *k*, in the treatment group *t*: if *n*_*jkt*_ is missing, one solution could be taking the minimal value between sample size *n*_*jt*_ that counts for individuals reporting outcome *j* and *n*_*kt*_ for outcome *k*. Although *n*_*jkt*_ may be inaccurately imputed, this solution could be better than removing the two effect sizes. As a model preparation package, metavcov could handle missing data problems more carefully by customizing functions for different types of effect sizes case by case. Moreover, the package also offers a function for multiple imputations for missing data, a compact computer program that is extensible for different estimation methods of users' choice.

### 1.1. Models

In general, an MMA specifies the model at within-study and between-study levels (Wei and Higgins, 2013b). For the within-study level, let ${\hat{\theta }}_{i}$ denote a vector of *p* observed effect sizes in the *i*th study, which is assumed from a multivariate normal distribution:

(1) $$\begin{array}{l} {\hat{\theta }}_{i}\sim \text{MVN}(\theta_{i},\Sigma_{i})\text{ with }\Sigma_{i}\\ \;=\left[\begin{array}{cccc} s_{i1}^{2} & \rho_{w12}s_{i1}s_{i2} & \cdots & \rho_{w1p}s_{i1}s_{ip}\\ \rho_{w21}s_{i1}s_{i2} & s_{i2}^{2} & \cdots & \rho_{w2p}s_{i2}s_{ip}\\ \vdots & \vdots & \ddots & \vdots \\ \rho_{wp1}s_{i1}s_{ip} & \rho_{wp2}s_{i2}s_{ip} & \cdots & s_{ip}^{2} \end{array}\right], \end{array}$$

where ***θ***_*i*_ is the vector of underlying true effect sizes for study *i* and **Σ**_*i*_ is the within-study variance-covariance matrix, which is composed of the sampling variance of each effect size on the diagonal, denoted by $s_{ij}^{2}$ (*j* = 1, …, *p*) for the *j*th effect size, and the within-study covariance of each pair of effect sizes on the off-diagonal that reflects within-study correlation, denoted by ρ_*wst*_ for the *s*th and *t*th effect sizes. Here, index *i* is omitted for ρ_*w*.._ for the reason of simplicity. In the next section, subscript *i* is added for each study, whereas subscript *w* is omitted for simplicity since the whole section is about within-study covariances. The assumption for ***θ***_*i*_ is that the sample is from a multivariate normal distribution that centers around the true effect sizes, denoted by $\theta ={\left(\theta_{1},\theta_{2},\ldots ,\theta_{p}\right)}^{T}$, as

$$\theta_{i}\sim \text{MVN}(\theta ,\Omega )\text{ with }\Omega =\left[\begin{array}{cccc} \tau_{1}^{2} & \rho_{b12}\tau_{1}\tau_{2} & \cdots & \rho_{b1p}\tau_{1}\tau_{p}\\ \rho_{b21}\tau_{1}\tau_{2} & \tau_{2}^{2} & \cdots & \rho_{b2p}\tau_{2}\tau_{p}\\ \vdots & \vdots & \ddots & \vdots \\ \rho_{bp1}\tau_{1}\tau_{p} & \rho_{bp2}\tau_{2}\tau_{p} & \cdots & \tau_{p}^{2} \end{array}\right],$$

where **Ω** is the between-study variance-covariance matrix, which is composed of between-study variance for each true effect size on the diagonal and between-study covariance for each pair of effect sizes on the off-diagonal that reflects between-study correlations ρ_*b*.._. This model can also be written as ${\hat{\theta }}_{i}\;\sim \text{ MVN}(\theta ,\Sigma_{i}+\Omega )$. By adding **Ω**, random effects between studies are accommodated. When **Ω** = **0**, the model is refered as a fixed effect model. For meta-regression, it is written as ***y***_*i*_ ~ MVN(***X***_*i*_***β***, **Σ**_*i*_ + **Ω**), where the notation of ${\hat{\theta }}_{i}$ is substituted by ***y***_*i*_ to follow the notation in regression models.

To fit a fixed/random effect meta-analysis or meta-regression, we have to calculate ${\hat{\theta }}_{i}$ and ${\hat{\Sigma }}_{i}$ for study *i* = 1, …, *N*. In practice, ${\hat{\Sigma }}_{i}$ is computed from formulas involving ${\hat{\theta }}_{i}$ to replace **Σ**_*i*_ in equation (1), which is discussed in the next section. Although most effect sizes and their variances/covariances in this articles refer to the estimated values, we omit the circumflex in their notations like other articles (Olkin, 1976; Wei and Higgins, 2013b; Borenstein et al., 2021) for the sake of simplicity. Alternatively, one could interpret those notations as the sample estimators from each study conforming to the same formulas as the true underlying random variables. For packages mixmeta and metaSEM, we have to prepare (1) a matrix **Θ**, which is an *N* by *p* matrix with ${\hat{\theta }}_{i}$ in each row and is contained via the argument data in both packages, and (2) a matrix **Ξ** which is an *N* by *p*(*p* + 1)/2 matrix that saves all the variances and covariances from ${\hat{\Sigma }}_{i}$ for study *i* in each row, denoted by ***S***_*i*_. Note that ${\hat{\Sigma }}_{i}$ is a *p* by *p* symmetric matrix with *p* + (*p* − 1) + (*p* − 2) ⋯ + 1 = *p*(*p* + 1)/2 unique elements. It is more convenient to store these unique elements in a vector ***S***_*i*_, which is organized as $S_{i}={\left(s_{i1}^{2},\rho_{w21}s_{i1}s_{i2},\ldots ,\rho_{wp1}s_{i1}s_{ip},s_{i2}^{2},\ldots ,\rho_{wp2}s_{i2}s_{ip},\ldots ,s_{ip}^{2}\right)}^{T}$ from the lower triagonal entries in ${\hat{\Sigma }}_{i}$. **Ξ** is contained in the argument v in the metaSEM package. For the mixmeta package, **Ξ** is contained in the argument S, and S also accepts an *N*-dimensional list of *p* × *p* matrices where ${\hat{\Sigma }}_{i}$ is stored.

This article describes how to estimate with-study variance-covariance matrix **Σ**_*i*_ in the next section with details including missing data solutions, where the notation $\hat{\theta }$ is replaced according to different types of effect sizes, such as *r* for correlation coefficients and δ for standardized mean differences. Furthermore, this article provides a model estimation section with a data visualization example and a section focusing on missing data problems with a simulation study. Package summary and future work are given in the end.

## 2. Estimating the with-study variance-covariance matrix **Σ**_*i*_

### 2.1. Correlation coefficient

Let *r*_*ist*_ denote the sample correlation coefficient that describes the relationship between variables *s* and *t* in study *i*. Following the notation by Olkin (1976), Becker (2009), and Ahn et al. (2015), we have

$$\text{var}\left(r_{ist}\right)={\left(1-\rho_{ist}^{2}\right)}^{2}/n_{i}$$

for the variance of *r*_*ist*_, and the covariance between *r*_*ist*_ and *r*_*iuv*_ is

(2) $$\begin{array}{l} \text{cov}\left(r_{ist},r_{iuv}\right)=[.5\rho_{ist}\rho_{iuv}\left(\rho_{isu}^{2}+\rho_{isv}^{2}+\rho_{itu}^{2}+\rho_{itv}^{2}\right)+\rho_{isu}\rho_{itv}\\ \;+\rho_{isv}\rho_{itu}\\ \;-(\rho_{ist}\rho_{isu}\rho_{isv}+\rho_{its}\rho_{itu}\rho_{itv}+\rho_{ius}\rho_{iut}\rho_{iuv}\\ \;+\rho_{ivs}\rho_{ivt}\rho_{ivu})]/n_{i}, \end{array}$$

where ρ_*i*.._ represents the corresponding population value. In practice, ρ_*i*.._ can be substituted by the observed sample correlation *r*_*i*.._ (Ahn et al., 2015), and var(*r*_*ist*_) and cov(*r*_*ist*_, *r*_*iuv*_) could be calculated by setting the argument method = "each" in the function r.vcov(). Note that the calculation of cov(*r*_*ist*_, *r*_*iuv*_) also involes *r*_*iut*_, *r*_*itv*_, …  that could be missing in the real data, which may make it impossible to conduct MMA for *r*_*ist*_ and *r*_*iuv*_. In this case, the argument method can be set as "average", so that sample-size weighted mean from all available studies can be chosen to replace ρ_*i*.._ in equation (2), which was proposed by Cooper et al. (2009). Furthermore, we can transform *r*_*ist*_ into the Fisher's *z* score as

$$z_{ist}=\frac{1}{2}\ln\;\left(\frac{1+r_{ist}}{1-r_{ist}}\right).$$

When Fisher's *z* scores are used, variances and covariances can be computed as

$$\begin{array}{c} \text{var}\left(z_{ist}\right)=1/\left(n_{i}-3\right)\text{and cov}\left(z_{ist},z_{iuv}\right)=\text{cov}\left(r_{ist},r_{iuv}\right)/\\ \left[\left(1-\rho_{ist}^{2}\right)\left(1-\rho_{iuv}^{2}\right)\right]. \end{array}$$

**Figure 2:**
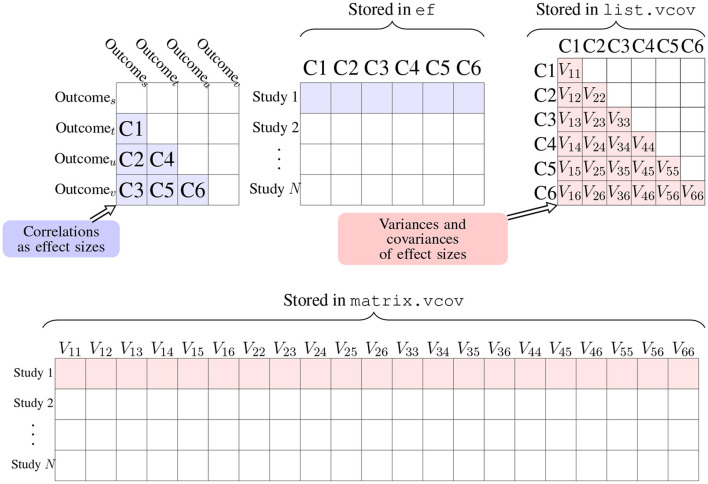
Arrangement of effect sizes and their covariances in matrix and list formats using correlation coefficients as an example. The output value list.vcov is a list of *N* matrices, in which list.vcov[[i]] represents the matrix **Σ**_*i*_ in Equation (1), where the element *V*_*jk*_ in the above figure equals to ρ_*wjk*_*s*_*ij*_*s*_*ik*_ in Equation (1) and *V*_*jj*_ equals to $s_{ij}^{2}$ as the variance of ${\hat{\theta }}_{ij}$. The output value matrix.vcov transforms list.vcov into an *N* × *p*(*p* + 1)/2 matrix. We could use ef and matrix.vcov as input arguments for packages mixmeta or metaSEM to fit an MMA model.

In addition to the arguments method and n as the sample size, the R function r.vcov() needs another argument corflat to input correlation coefficients from studies as an *N* by *p* matrix where values of *r*_*ist*_ are saved in each row. The computed *z* scores are saved in the output value ef, which is an *N* by *p* matrix in the same format of argument corflat shown in Figure 2 in blue. From r.vcov(), the output values ef, list.vcov, and matrix.vcov are calculated Fisher's *z* scores and their covariances; the corresponding values in the scale of Pearson's correlation coefficients are stored in output values r, list.rvcov, and marix.rvcov. In the next subsections, the function mix.vcov() can be used for other effect sizes, which also provides output values ef, list.vcov and matrix.vcov.

From r.vcov(), the output value list.rvcov is a list of *N* matrices, in which list.rvcov[[i]] stores var(*r*_*ist*_) and cov(*r*_*ist*_, *r*_*iuv*_) in equation (2) for study *i*. The following shows the example from Cooper et al. (2009) on page 388 as an illustration.


r  <- matrix(c(-0.074, -0.127, 0.324, 0.523, -0.416, -0.414), 1)
n  <- 142
computvcov  <- r.vcov(n = n, corflat = r,
                             name = paste("C",  c("st", "su",  "sv",
                                    "tu", "tv", "uv"),  sep = ""),
                             method = "each")
round(computvcov$list.rvcov[[1]], 4)
      Cst     Csu     Csv     Ctu     Ctv     Cuv
      Cst  0.0070  0.0036 -0.0025 -0.0005  0.0018  0.0009
      Csu  0.0036  0.0068 -0.0025 -0.0002  0.0008  0.0017
      Csv -0.0025 -0.0025  0.0056  0.0001  0.0000 -0.0003
      Ctu -0.0005 -0.0002  0.0001  0.0037 -0.0013 -0.0013
      Ctv  0.0018  0.0008  0.0000 -0.0013  0.0048  0.0022
      Cuv  0.0009  0.0017 -0.0003 -0.0013  0.0022  0.0048


The *z* transformed correlation coefficients are saved in the output vector ef.


round(computvcov$ef, 4)
         Cst     Csu     Csv    Ctu     Ctv    Cuv
      1 -0.0741 -0.1277 0.3361 0.5805 -0.4428 -0.4404

round(computvcov$list.vcov[[1]], 4)
      Cst     Csu     Csv     Ctu     Ctv     Cuv
      Cst  0.0072  0.0037 -0.0029 -0.0008  0.0022  0.0011
      Csu  0.0037  0.0072 -0.0028 -0.0003  0.0010  0.0021
      Csv -0.0029 -0.0028  0.0072  0.0001  0.0000 -0.0004
      Ctu -0.0008 -0.0003  0.0001  0.0072 -0.0022 -0.0022
      Ctv  0.0022  0.0010  0.0000 -0.0022  0.0072  0.0032
      Cuv  0.0011  0.0021 -0.0004 -0.0022  0.0032  0.0072


Note that for *m* outcomes, there are *p* = *m* × (*m* − 1)/2 correlation coefficients. Since the *p* by *p* variance-covariance matrix is symmetric, there are *p* + (*p* − 1) + (*p* − 2) ⋯ + 1 = *p*(*p* + 1)/2 unique elements. It is more convenient to store these unique elements in a vector so that if we have *N* studies, we could have an *N* by *p*(*p* + 1)/2 matrix that saves all the variances and covariances, which can be obtained from the output value matrix.vcov. The bottom row in Figure 2 is an illustration of how the variances and covariances are arranged in matrix and list formats. Following the above code, we have

For missing values, we could impute a numeric value such as zero via the argument na.impute.


computvcov  <- r.vcov(n = 142,
  corflat = matrix(c(-0.074, -0.127, 0.324,
  0.523, -0.416, NA), 1),
                     na.impute = 0)



round(computvcov$matrix.vcov, 4)
           var_Cst cov_Cst_Csu cov_Cst_Csv cov_Cst_Ctu
      [1,]  0.0072      0.0037     -0.0029     -0.0008
           cov_Cst_Ctv cov_Cst_Cuv var_Csu cov_Csu_Csv
      [1,]      0.0022      0.0011  0.0072     -0.0028
           cov_Csu_Ctu cov_Csu_Ctv cov_Csu_Cuv var_Csv
      [1,]     -0.0003      0.0010      0.0021  0.0072
           cov_Csv_Ctu cov_Csv_Ctv cov_Csv_Cuv var_Ctu
      [1,]      0.0001   0.0000     -0.0004   0.0072
           cov_Ctu_Ctv cov_Ctu_Cuv var_Ctv  cov_Ctv_Cuv   var_Cuv
      [1,]     -0.0022     -0.0022  0.0072      0.0032     0.0072



computvcov$r
      C1     C2     C3    C4    C5     C6
1 -0.074 -0.127 0.324 0.523 -0.416  0


By default, we have na.impute = NA without any imputation. Under the default setting of method = "average", the calculation of cov(*r*_*ist*_, *r*_*iuv*_) is still possible even though it involes *r*_*iut*_, *r*_*itv*_, …  that could be missing. In addition to imputing a specific number via na.impute, we could also impute the sample-size-weighted mean from those studies with complete records by setting the argument na.impute = "average". Basically, na.impute = "average" imputes the mean values for *r*_*ist*_, *z*_*ist*_ and cov(*r*_*ist*_, *r*_*iuv*_), while method = "average" imputes the mean values only for cov(*r*_*ist*_, *r*_*iuv*_). These two arguments, na.impute = "average" and method = "average", match the mean imputation method and the method of omission illustrated in Section 4.2 for the missing data problem. Note that all the discussion about missing data in Sections 2 and 3 is about missingness in within-study factors. Missingness in between-study factors can only be handled in functions described in Section 4.

### 2.2. Standardized mean difference

For the treatment group, let *n*_*jt*_, *n*_*kt*_, and *n*_*jkt*_ denote the numbers of participants who report outcome *j*, *k*, and both outcomes *j* and *k*, respectively. Similarly, denote *n*_*jc*_, *n*_*kc*_, and *n*_*jkc*_ for the control group. These notations are used for all the effect sizes for treatment comparison, including standardized mean difference (SMD), mean difference, log odds ratio, log risk ratio, and risk difference. There are two ways to estimate the population SMD, Hedges' *g* and the sample SMD. Denote the sample mean score on outcome *j* in the treatment and control groups as ȳ_*jt*_ and ȳ_*jc*_, respectively, and the standard deviation of the scores as *s*_*jt*_ and *s*_*jc*_. Hedges (1981) proposed a minimum variance unbiased estimator for the population SMD, which is defined as


$$g_{j}=\frac{\delta_{j}}{J\left(v_{j}\right)}\text{ with }J\left(v_{j}\right)=\frac{\Gamma \left(v_{j}/2\right)}{\sqrt{\frac{v_{j}}{2}\Gamma \left(\frac{v_{j}-1}{2}\right)}}\text{ and }v_{j}=n_{jt}+n_{jc}-2,$$

where


$$\delta_{j}=\frac{{ȳ}_{jt}-{ȳ}_{jc}}{s_{j}^{\text{pool}}}\text{ with }s_{j}^{\text{pool}}=\sqrt{\frac{\left(n_{jt}-1\right)s_{jt}^{2}+\left(n_{jc}-1\right)s_{jc}^{2}}{n_{jt}+n_{jc}-2}}.$$

Wei and Higgins (2013b) derived the covariance between two effect sizes in terms of Hedges' *g*, denoted by *g*_*j*_ and *g*_*k*_, as follows


$$\begin{array}{l} \text{cov}\left(g_{j},g_{k}\right)=\rho \left(\frac{n_{jkc}}{n_{jc}n_{kc}}+\frac{n_{jkt}}{n_{jt}n_{kt}}\right)+\frac{k_{jk}}{k_{j}k_{k}}\rho^{2}\delta_{j}\delta_{k}J\left(v_{j}\right)J\left(v_{k}\right)\\ \;\sqrt{\left(\frac{v_{j}}{v_{j}-2}-\frac{1}{{J\left(v_{j}\right)}^{2}}\right)\left(\frac{v_{k}}{v_{k}-2}-\frac{1}{{J\left(v_{k}\right)}^{2}}\right)},\\ \text{where }k_{k}=\frac{2n_{kt}+2n_{kc}-4}{{\left(n_{kc}+n_{kt}-2\right)}^{2}},\\ \;k_{j}=\frac{2n_{jt}+2n_{jc}-4}{{\left(n_{jc}+n_{jt}-2\right)}^{2}},\\ \;k_{jk}=\frac{2}{\left(n_{jc}+n_{jt}-2\right)\left(n_{kc}+n_{kt}-2\right)}\\ \;\left(\frac{n_{jt}n_{kt}}{n_{jt}+n_{kt}-1}+\frac{n_{jc}n_{kc}}{n_{jc}+n_{kc}-1}-2\right), \end{array}$$

and ρ is a simplified notation of ρ_*wjk*_ in Equation (1).

We could use the function smd.vcov() for calculating Hedges' g from SMD, which is stored in the output value ef. The input arguments for δ_*j*_, *n*_*jt*_, and *n*_*jc*_ are d, nt, and nc which are all *N* × *p* matrices in the same arrangement as ef in Figure 2. The arguments for ρ, *n*_*jkt*_, and *n*_*jkc*_ are r, n_rt, and n_rc which are all in a list format with *N* *p* × *p* matrices. If *n*_*jkt*_ or *n*_*jkc*_ is missing, the function automatically imputes *n*_*jkt*_ by the minimal value between *n*_*jt*_ and *n*_*kt*_, and imputes *n*_*jkc*_ by the minimal value between *n*_*jc*_ and *n*_*kc*_. This imputation method is used for all the effect sizes for treatment comparison, including SMD, mean difference, log odds ratio, log risk ratio, and risk difference. The variances and covariances of Hedges' g are stored in matrix.vcov and list.vcov in the same arrangement shown in the bottom row of Figure 2.

The function smd.vcov() also provides the formula in Olkin and Gleser (2009) for the covariance of the sample SMD, δ_*j*_, which is defined as


$$\text{cov}\left(\delta_{j},\delta_{k}\right)=\frac{\left(n_{t}+n_{c}\right)\rho }{n_{t}n_{c}}+\frac{\delta_{j}^{}\delta_{k}^{}\rho^{2}}{2\left(n_{t}+n_{c}\right)}.$$

The results are stored in the output values matrix.dvcov and list.dvcov in the same formats of matrix.vcov and list.vcov, respectively. To demonstrate the usage of smd.vcov(), the dataset in Geeganage and Bath (2010) is applied using variables SMD_SBP and SMD_DBP, which measure the systolic blood pressure (SBP, in mHg) and diastolic blood pressure (DBP, in mHg). The correlation between SBP and DBP is not recorded in the article, so we impute it as 0.71 based on expert knowledge — ideally, different correlation coefficients should be recorded from *N* different primary studies saved in a list of *N* correlation matrices.


data(Geeganage2010)
## correlation coefficients between outcomes are missing in the data
## impute the correlation coefficient list based on expert knowledge
r12  <- 0.71
r.Gee  <- lapply(1:nrow(Geeganage2010),
                     function(i){matrix(c(1, r12, r12, 1), 2, 2)})

computvcov  <- smd.vcov(nt = Geeganage2010[,c("nt_SBP", "nt_DBP")],
                        nc = Geeganage2010[,c("nc_SBP", "nc_DBP")],
                       d = Geeganage2010[,c("SMD_SBP", "SMD_DBP")],
                       r = r.Gee,
                       name = c("SBP", "DBP"))
head(computvcov$ef)   ## Hedge's g
          SBP           DBP
      1 -0.075002006 -0.19339306
      2  0.043155405 -0.01610660
      3 -0.242782681 -0.31689607
      4 -0.097028863 -0.16608808
      5 -0.004460966 -0.13364520
      6 -0.286780271  0.08887979
head(computvcov$matrix.vcov)  ## variances/covariances for Hedge's g
           var_SBP     cov_SBP_DBP   var_DBP
      [1,] 0.15560955  0.11051462 0.15591453
      [2,] 0.18256182  0.12959901 0.18254277
      [3,] 0.03190808  0.02264927 0.03198210
      [4,] 0.03115906  0.02212545 0.03119080
      [5,] 0.01965510  0.01395547 0.01967717
      [6,] 0.26813782  0.18910349 0.26680797

head(computvcov$matrix.dvcov)  ## variances/covariances for SMD
           var_SBP     cov_SBP_DBP   var_DBP
      [1,] 0.15565024  0.11056752 0.15618509
      [2,] 0.18257730  0.12959610 0.18254492
      [3,] 0.03200824  0.02271517 0.03215273
      [4,] 0.03117474  0.02213897 0.03123674
      [5,] 0.01965512  0.01395583 0.01969852
      [6,] 0.26896403  0.18897441 0.26688733


### 2.3. Mean difference and log odds ratio

Sometimes researchers prefer to keep the original scale of mean differences (MD) instead of standardizing them into SMD, such as body mass index (BMI) (Torloni et al., 2009; Winter et al., 2014) or waist circumference (Czernichow et al., 2011; de Hollander et al., 2012). For dichotomous outcomes such as mortality or morbidity, a popular effect size measurement is the log odds ratio (logOR) (Insua et al., 1994; Thompson et al., 1997). Following the notations for SMD, Wei and Higgins (2013b) also derived the covariances for MD and logOR as

$$\text{cov}\left({\text{MD}}_{j},{\text{MD}}_{k}\right)=\frac{n_{jkt}}{n_{jt}n_{kt}}\rho s_{jt}s_{kt}+\frac{n_{jkc}}{n_{jc}n_{kc}}\rho s_{jc}s_{kc}$$

and

$$\begin{array}{l} \text{cov}\left({\text{logOR}}_{j},{\text{log OR}}_{k}\right)=\frac{\rho n_{jkc}}{n_{jc}n_{2c}}\sqrt{\left(\frac{1}{S_{jc}}+\frac{1}{F_{jc}}\right)\left(\frac{1}{S_{kc}}+\frac{1}{F_{kc}}\right)}\\ \;+\frac{\rho n_{jkt}}{n_{jt}n_{kt}}\sqrt{\left(\frac{1}{S_{jt}}+\frac{1}{F_{jt}}\right)\left(\frac{1}{S_{kt}}+\frac{1}{F_{kt}}\right)}, \end{array}$$

where *S*_*jt*_ and *S*_*jc*_ are the numbers of participants with the outcome *j* event in the treatment and control groups, respectively, and *F*_*jt*_ and *F*_*jc*_ are the respective numbers without the event. Functions md.vcov() and logOR.vcov() can be used to calculate cov(MD_*j*_, MD_*k*_) and cov(logOR_*j*_, logOR_*k*_). Similar to r.vcov() and smd.vcov(), the variance-covariance matrices are stored in the output values matrix.vcov and list.vcov in matrix and list formats, and the calculated log odds ratios are stored in the output value ef. Similar functions in the **metavcov** package include lgRR.vcov() for log risk ratios and rd.vcov() for risk differences. The function mix.vcov() is designed for merging all of these functions whose details are demonstrated in the next subsection.

The covariance between MD and logOR is calculated as

$$\begin{array}{l} \text{cov}\left({\text{MD}}_{j},{\text{logOR}}_{k}\right)=\rho s_{jc}\frac{n_{jkc}\sqrt{n_{kc}}}{n_{jc}n_{kc}}\sqrt{\left(\frac{1}{S_{kc}}+\frac{1}{F_{kc}}\right)\left(\frac{1}{S_{kc}}+\frac{1}{F_{kc}}\right)}\\ \;+\rho s_{jt}\frac{n_{jkt}\sqrt{n_{kt}}}{n_{jt}n_{kt}}\sqrt{\left(\frac{1}{S_{kt}}+\frac{1}{F_{kt}}\right)\left(\frac{1}{S_{kt}}+\frac{1}{F_{kt}}\right)}, \end{array}$$

which can be obtained using the function md_lgor(), whose output values include lgor that returns the computed log odds ratio and v that returns the computed covariance.


md_lgor(r = 0.71, sd1t = 0.4, sd1c = 8,
      n1c = 34, n2c = 35,
      n1t = 25, n2t = 32,
      s2c = 5, s2t = 8,
      f2c = 30, f2t = 24)
$lgor  ## computed log odds ratio (logOR)
      [1] 0.6931472
$v ## computed covariance between the
MD and logOR
      [1] 0.484266


### 2.4. Combination of effect sizes

**Figure 3:**
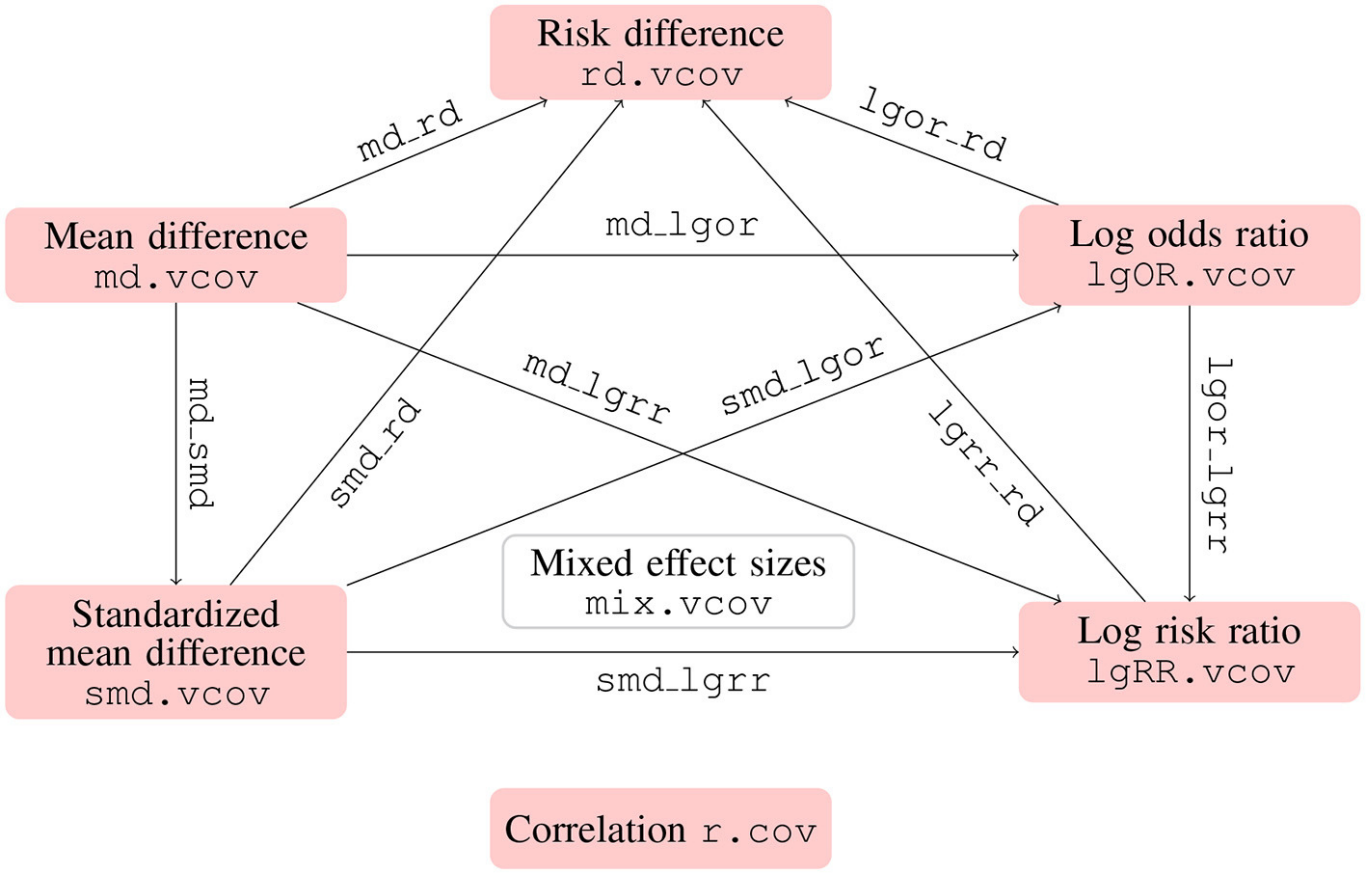
Functions for computing covariances between different types of effect sizes using metavcov. Functions with names connected by dot compute effect sizes of the same type (except mix.vcov) and work with multiple studies, while functions with names connected by underscore compute two effect sizes of different types and only work with one study for simple calculation.

In addition to the correlation coefficients, SMD, MD, and logOR, the metavcov package also includes log risk ratio (logRR) and risk difference (RD). The formulas for calculating their covariances can be found in Table 1 in Wei and Higgins (2013b) and the corresponding R functions can be found in Figure 3. Similar to the function md_lgor() in the previous subsection, we have lgor_lgrr() for covariance between logOR and logRR, lgor_rd() for covariance between logOR and RD, md_lgrr() for covariance between MD and logRR, md_rd() for covariance between MD and RD, md_smd() for covariance between MD and SMD, smd_lgor() for covariance between SMD and logOR, smd_lgrr() for covariance between SMD and logRR, and smd_rd() for covariance between SMD and RD. These functions are designed for simple calculations to prepare for the function mix.vcov(), which merges all of these functions by specifying the input argument type with options "MD" for mean difference, "SMD" for standardized mean difference, "logOR" for log odds ratio, "logRR" for log risk ratio, and "RD" for risk difference. Its output values ef, matrix.vcov, and list.vcov are the calculated effect sizes and covariances in matrix and list formats.

To demonstrate the usage of mix.vcov(), the dataset in Geeganage and Bath (2010) is applied again. There are four outcomes, including systolic blood pressure (SBP, in mHg), diastolic blood pressure (DBP, in mHg), death (D), and death or disability (DD). Mean difference is used to measure the two continuous outcomes SBP and DBP. Risk difference and log odds ratio are chosen to measure the two dichotomous outcomes D and DD. The type of their effect sizes is specified via a vector for argument type in order. This order is applied to all the other arguments. Note that certain arguments are not available for specific outcomes. For example, arguments d, sdt, and sdc are designed for effect sizes SMD or MD, which are not available for logOR, logRR, or RD. Therefore, we have to impute NAs in arguments d, sdt, and sdc for outcomes D and DD. Similarly, we have to impute NAs for st and sc for outcomes SBP and DBP. The correlation coefficients between these outcomes are not recorded in the article, so we impute them based on expert knowledge—ideally, different correlation coefficients should be recorded from *N* different primary studies saved in a list of *N* correlation matrices. The example code is as follows.


data(Geeganage2010)
## correlation coefficients between outcomes are missing in the data
## impute the correlation coefficient list based on expert knowledge
r12  <- 0.71
r13  <- 0.5
r14  <- 0.25
r23  <- 0.6
r24  <- 0.16
r34  <- 0.16
r  <- vecTosm(c(r12, r13, r14, r23, r24, r34))
diag(r)  <- 1
mix.r  <- lapply(1:nrow(Geeganage2010), function(i){r})
attach(Geeganage2010)
computvcov  <- mix.vcov(type = c("MD", "MD", "RD", "lgOR"),
                         d = cbind(MD_SBP, MD_DBP, NA, NA),
                         sdt = cbind(sdt_SBP, sdt_DBP, NA, NA),
                         sdc = cbind(sdc_SBP, sdc_DBP, NA, NA),
                         nt = cbind(nt_SBP, nt_DBP, nt_DD, nt_D),
                         nc = cbind(nc_SBP, nc_DBP, nc_DD, nc_D),
                         st = cbind(NA, NA, st_DD, st_D),
                         sc = cbind(NA, NA, sc_DD, sc_D),
                         
 
r = mix.r,
               name = c("MD.SBP","MD.DBP","RD.DD","lgOR.D"))
## save different effect sizes in y
y  <- computvcov$ef
head(y)
          MD.SBP  MD.DBP     RD.DD    lgOR.D
        1  -2.47  -3.44  0.00000000 -1.0986123
        2   1.61  -0.34  0.18750000  0.5959834
        3  -8.16  -6.44  0.02554455  0.5892102
        4  -3.17  -3.41  0.04000000  0.4444945
        5  -0.15  -2.39  0.01920750  0.1000835
        6  -9.83   1.93 -0.25000000 -0.5108256
 
computvcov$list.vcov[[1]]
                 MD.SBP     MD.DBP      RD.DD     lgOR.D
        MD.SBP 87.9883122 34.8140903 0.92452778 2.27820442
        MD.DBP 34.8140903 27.8514100 0.62070000 0.79071907
        RD.DD   0.9245278  0.6207000 0.04062500 0.02741618
        lgOR.D  2.2782044  0.7907191 0.02741618 1.02083333

$# save variances/covariances of all the effect sizes in a matrix S
S  <- computvcov$matrix.vcov
S[1, ]
  var_MD.SBP cov_MD.SBP_MD.DBP   cov_MD.SBP_RD.DD  cov_MD.SBP_lgOR.D
 1   87.98831        34.81409         0.9245278       2.278204
  var_MD.DBP   cov_MD.DBP_RD.DD  cov_MD.DBP_lgOR.D  var_RD.DD
 1   27.85141          0.6207          0.7907191      0.040625
  cov_RD.DD_lgOR.D   var_lgOR.D
 1   0.02741618      1.020833


**Figure 4:**
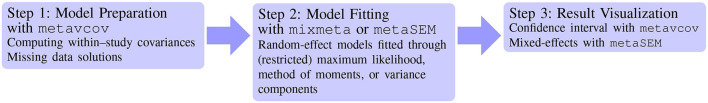
Illustration of the workflow for conducting MMA using the R packages introduced.

The matrices y and S in the above code can be used as input arguments for packages mixmeta and metaSEM, which is demonstrated in the next section. After computing within-study covariances, the next step is model fitting for estimating the overall effect sizes, potentially with result visualizations (see Figure 4).

## 3. Estimating the overall effect sizes

### 3.1. Generalized least squares method

The GLS method (Berkey et al., 1996) enables us to estimate the overall effect size ***θ*** from the observed ${\hat{\theta }}_{i}$ and **Σ**_*i*_ from all the *N* studies. It is similar to the more familiar ordinary least squares method, but it allows the data from which parameters are estimated to have unequal population variances and nonzero covariances. Becker (2009) have shown that the GLS estimators are also maximum likelihood estimators. This section demonstrates the GLS procedure in order that the next section could present handling the missing data problem under its framework.

First, let $T_{Np\times 1}=({\hat{\theta }}_{11},{\hat{\theta }}_{12},\ldots ,{\hat{\theta }}_{1p},{\hat{\theta }}_{21},{\hat{\theta }}_{22},\ldots ,{\hat{\theta }}_{2p},\ldots ,$ ${\hat{\theta }}_{i1},{\hat{\theta }}_{i2},\ldots ,{\hat{\theta }}_{ip},\ldots ,{\hat{\theta }}_{N1},{\hat{\theta }}_{N2},\ldots ,{\hat{\theta }}_{Np}{)}^{\prime }$ be a rearrangement of elements in ${\hat{\theta }}_{i}$ from all the *N* studies. Given an error vector, denoted by ***e***_*Np*×1_, the relationship between the population parameter $\theta ={\left(\theta_{1},\theta_{2},\ldots ,\theta_{p}\right)}^{\prime }$ and ***T*** is

(3) $$\begin{array}{l} T_{Np\times 1}=X_{Np\times p}\theta +e_{Np\times 1}=\left[\begin{array}{cccccc} 1 & 0 & 0 & \cdots & 0 & 0\\ 0 & 1 & 0 & \cdots & 0 & 0\\ \vdots & \vdots & \ddots & \vdots & \vdots & \vdots \\ 0 & 0 & \cdots & 1 & \cdots & 0\\ \vdots & \vdots & \vdots & \vdots & \ddots & \vdots \\ 0 & 0 & \cdots & 0 & 0 & 1\\ \; & \; & \vdots & \vdots \\ 1 & 0 & 0 & \cdots & 0 & 0\\ 0 & 1 & 0 & \cdots & 0 & 0\\ \vdots & \vdots & \ddots & \vdots & \vdots & \vdots \\ 0 & 0 & \cdots & 1 & \cdots & 0\\ \vdots & \vdots & \vdots & \vdots & \ddots & \vdots \\ 0 & 0 & \cdots & 0 & 0 & 1 \end{array}\right]\left[\begin{array}{c} \theta_{1}\\ \theta_{2}\\ \vdots \\ \theta_{j}\\ \vdots \\ \theta_{p} \end{array}\right]+\left[\begin{array}{c} e_{11}\\ e_{12}\\ \vdots \\ e_{1j}\\ \vdots \\ e_{1p}\\ \vdots \\ e_{N1}\\ e_{N2}\\ \vdots \\ e_{Nj}\\ \vdots \\ e_{Np} \end{array}\right], \end{array}$$

where ***X*** is an *Np* × *p* matrix created by stacking *N* *p*-dimensional identity matrices.

Assuming the errors in ***e*** are normally distributed with a zero mean vector **0** and a variance-covariance matrix **Ψ**, which is a blockwise diagonal matrix with **Σ**_*i*_ in its diagonal:

$$\Psi =\left[\begin{array}{cccccc} \Sigma_{1} & 0 & 0 & \cdots & 0 & 0\\ 0 & \Sigma_{2} & 0 & \cdots & 0 & 0\\ \vdots & \vdots & \ddots & \vdots & \vdots & \vdots \\ 0 & 0 & \cdots & \Sigma_{i} & \cdots & 0\\ \vdots & \vdots & \vdots & \vdots & \ddots & \vdots \\ 0 & 0 & \cdots & 0 & 0 & \Sigma_{N} \end{array}\right].$$

Note that in a random effect model, the matrix in its diagonal is **Σ**_*i*_ + **Ω**.

The GLS estimator of ***θ*** and its variance $\text{Var}\left(\hat{\theta }\right)$ are given by

(4) $$\begin{array}{l} \hat{\theta }={\left(X^{\prime }\Psi^{-1}X\right)}^{-1}X^{\prime }\Psi^{-1}T\text{ and Var}\left(\hat{\theta }\right)={\left(X^{\prime }\Psi^{-1}X\right)}^{-1}. \end{array}$$

A test of homogeneity with the null hypothesis *H*_0_: θ_1_ = θ_2_ = ⋯ = θ_*j*_ = ⋯ = θ_*p*_ can be conducted via the *Q* statistic (Higgins and Thompson, 2002; Sera et al., 2019):

$$Q={\hat{\theta }}^{\prime }\left[\Psi^{-1}-\Psi^{-1}X{\left(X^{\prime }\Psi^{-1}X\right)}^{-1}X^{\prime }\Psi^{-1}\right]\hat{\theta },$$

which follows a Chi-square distribution with df = (*N* − 1) × *p* degrees of freedom. The *Q* statistic generates the *I*^2^ statistic,

$$I^{2}=\max\left\{\frac{Q-\text{df}}{Q},0\right\},$$

which quantifies the amount of heterogeneity as the proportion of total variation related to sampling error. A value of 0% indicates no observed heterogeneity, and larger values show increasing heterogeneity (Higgins et al., 2003). We can use the function metafixed() for conducting a fixed-effect MMA, which is equivalent as setting method = "fixed" in mixmeta() using the mixmeta package. However, the zero heterogeneity fixed effect model is almost never appropriate for psychology.

For random effect models, methods including the maximum likelihood and the restricted maximum likelihood methods (Harville, 1977; Gasparrini et al., 2012; Sera et al., 2019), the method of moments (Chen et al., 2012; Jackson et al., 2013), and the method of two stages proposed by Liu et al. (2009) can be used to estimate **Ω** and ***θ***. These methods can be adopted in the mixmeta package by specifying method as "ml", "reml", "mm", or "vc" in mixmeta(). The metaSEM package adopts the maximum likelihood and the full information maximum likelihood methods (Cheung, 2021) in functions meta() and metaFIML(), respectively. When the effect sizes of interest are correlation coefficients, we can use metaSEM for conducting meta-analytic structural equation modeling (Cheung, 2008, 2009, 2013, 2015). A simple example for the metaSEM package is demonstrated as follows using y and S obtained via the output values ef and matrix.vcov from the previous code. For the maximum likelihood estimation method, we have


library(metaSEM)
MMA_RE  <- summary(meta(y = y, v = S,
data = data.frame(y,S)))


For the restricted maximum likelihood (REML) estimation method, we have


library(metaSEM)
MMA_RE  <- summary(reml(y = y, v = S,
data = data.frame(y,S)))


The argument data in the above functions is unnecessary. This is to show that functions mixmeta(), meta(), and reml() have the argument data so that covariates or predictors can be added for meta-regression.

In summary, we can use the function r.vcov() for correlation coefficients and mix.vcov() for other effect sizes from the metavcov package to calculate effect sizes and covariances, which are stored in output values ef and matrix.vcov. Then, we can use ef and matrix.vcov to conduct a random effect MMA via mixmeta or metaSEM. Note that regardless of the chosen function, estimating the full variance-covariance matrix **Ω** can be difficult unless *N* is large, because there are many parameters involved. Therefore, it is often wise to consider constrained models for the variance-covariance matrix **Ω** (McShane and Böckenholt, 2022).

### 3.2. Data visualization

The new version of metavcov offers a plot function plotCI() for displaying confidence intervals of effect sizes from each study and the overall estimators. The difference between a forest plot and a confidence interval plot is that a forest plot requires a symbol on each confidence interval that is proportional to the weight for each study (Schwarzer, 2007; Boyles et al., 2011; Sedgwick, 2015; Rücker and Schwarzer, 2021). Because the weighting mechanism in MMA is too complex to be visualized, such a proportional symbol is omitted. Although a confidence interval plot does not directly reflect weights for each study, it could provide sufficient information for users because effect sizes with narrower confidence intervals often contribute more to the overall estimators. Following the code from the previous subsection, an example for the function plotCI() is given below.


obj  <- MMA_FE
plotCI(y = computvcov$ef, v = computvcov$list.vcov,
       name.y = c(
        "Correlation: cognitive anxiety & somatic anxiety\n(C1)",
        "Correlation: cognitive anxiety & self concept\n(C2)",
        "Correlation: cognitive anxiety & athletic performance\n(C3)",
        "Correlation: somatic anxiety & self concept\n(C4)",
        "Correlation: somatic anxiety & athletic performance\n(C5)",
        "Correlation: self concept & athletic performance\n(C6)"),
       name.study = Craft2003$ID,
       y.all = obj$coefficients[,1],
       y.all.se = obj$coefficients[,2],
       up.bound = Inf, low.bound = -Inf)


**Figure 5:**
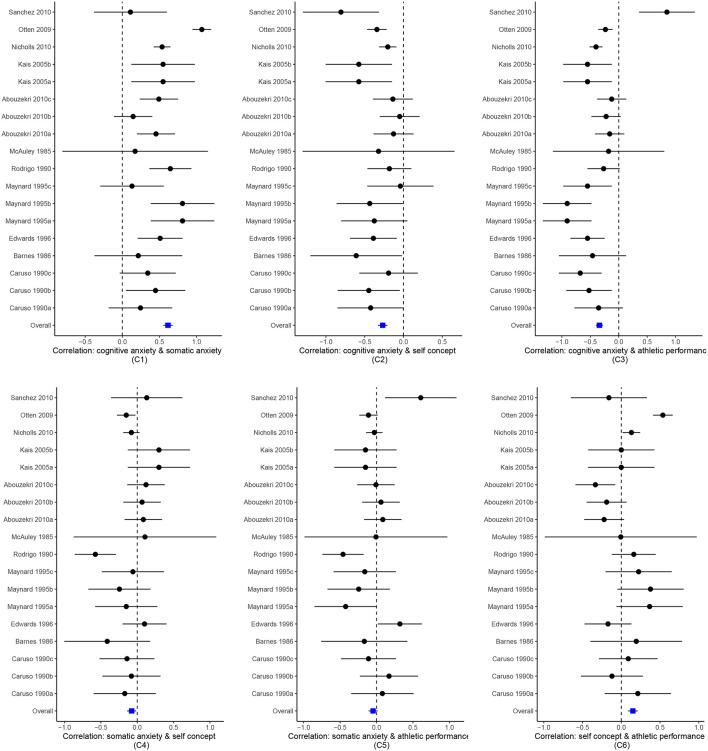
Confidence interval plots for effect sizes from the Craft et al. (2003) meta-analysis.

We could also set obj <- MMA_RE in the above code where MMA_RE was sepecified in the previous subsection from a random effect model using the package mixmeta or metaSEM. The result is shown in Figure 5.

## 4. The missing data problem

We can conveniently specify the predictors or missing values uing the design matrix ***X*** in Equation (3). First, let ***X*** be informally denoted as ***X*** = (𝕏(1), 𝕏(2), …, 𝕏(*i*), …, 𝕏(*N*))′ for simplicity, where 𝕏(*i*) is a *p*-dimensional identidy matrix in Equation (3). If we want to fit a meta-regression model (Van Houwelingen et al., 2002) with covariates or predictors *x*_*i*1_, *x*_*i*2_…  from each study, we can rewrite 𝕏(*i*) as

$$𝕏\left(i\right)=\left[\begin{array}{ccccccccc} 1 & 0 & 0 & \cdots & 0 & 0 & x_{i1} & x_{i2} & \ldots \\ 0 & 1 & 0 & \cdots & 0 & 0 & x_{i1} & x_{i2} & \ldots \\ \vdots & \vdots & \ddots & \vdots & \vdots & \vdots & \vdots & \vdots & \vdots \\ 0 & 0 & \cdots & 1 & \cdots & 0 & x_{i1} & x_{i2} & \ldots \\ \vdots & \vdots & \vdots & \vdots & \ddots & \vdots & \vdots & \vdots & \vdots \\ 0 & 0 & \cdots & 0 & 0 & 1 & x_{i1} & x_{i2} & \ldots \end{array}\right],$$

and ***θ*** as ${\left(\beta_{01},\beta_{02},\ldots ,\beta_{0p},\beta_{11},\beta_{12},\ldots ,\beta_{1p},\beta_{21},\beta_{22},\ldots ,\beta_{2p},\ldots \;\right)}^{\prime }$. In R, we could use mixmeta or metaSEM to conduct meta-regression. Following the code from the previous section, we could use the code below assuming the predictor is the percentage of male participants in each study.


summary(mixmeta(cbind(C1, C2, C3, C4, C5, C6)
~ p_male, S = S,
              data = data.frame(y,p_male
 = Craft2003$p_male),
              method = "reml"))


For the missing data problem, if the *j*th observed effect size is missing in study *i*, we could simply delete the *j*th row in 𝕏(*i*). For example, we expect C1, C2, … , C6 to be observed for all studies in the Craft et al. (2003) meta-analysis, where 𝕏(*i*) = ***I***_6_ for ***X*** in Equation (3). However, if C5 is missing in study *i*, then we could input

$$𝕏\left(i\right)=\left[\begin{array}{cccccc} 1 & 0 & 0 & 0 & 0 & 0\\ 0 & 1 & 0 & 0 & 0 & 0\\ 0 & 0 & 1 & 0 & 0 & 0\\ 0 & 0 & 0 & 1 & 0 & 0\\ 0 & 0 & 0 & 0 & 0 & 1 \end{array}\right]$$

in the design matrix ***X***. This method of omission for missing values in MMA is different from other regression functions such as those performed through lm() or glm() in R. Specifically, partially missing outcomes do not prevent the study from contributing to estimation. In addition to this omission procedure, we can also impute missing data by zero or the sample-size-weighted mean of observed effect sizes. Another way is to integrate the missing pattern in the estimation method such as Higgins et al. (2008) two-stage method or methods employing a Bayesian framework (Sutton et al., 2000; Yuan and Little, 2009). Although these techniques are not yet available for the current package, they may well become the methods of choice in future. Single imputation for missing effect sizes is available through the input argument na.impute in functions r.vcov() and mix.vcov(). Another option is multiple imputation.

### 4.1. Multiple imputation

Multiple imputation (MI) is a general approach that allows for the uncertainty about the missing data by generating several different plausible imputed datasets and appropriately combining results obtained from each of them (Allison, 2001; Schafer and Graham, 2002; Graham, 2009; Mavridis and Salanti, 2013; Little and Rubin, 2019). There are three basic phases for MI:

1. Imputation Phase: The missing data are imputed from simulated values drawn from some distributions. This process is repeated *M* times.
2. Analysis Phase: The same analysis is performed for each of the *M* complete datasets.
3. Pooling Phase: The *M* results are pooled to obtain the final result in some fashion.

Since MI methods involve recalculating the variance-covariance matrices for the studies with missing values, the new version of metavcov includes a function metami() that can conduct MI automatically. For the imputation phase, this function imports the package mice published by Van Buuren and Groothuis-Oudshoorn (2011) that imputes incomplete multivariate data by chained equations. The mice package is also recommended by the metafor package for univariate meta-analysis Viechtbauer (2021). For the analysis phase, all the functions mentioned in the previous section are accommodated, including metafixed(), mixmeta(), and meta(). The pooling phase is performed via Rubin's rules (Rubin, 1987; Barnard and Rubin, 1999; Van Buuren and Groothuis-Oudshoorn, 2011). Let ${\hat{\theta }}_{*m}$ be the estimated coefficient from the *m*th imputed dataset for one of the *p* dimensions in ***θ***, where *m* = 1, …, *M*. The pooled coefficient from MI, denoted by $\bar{\theta }$, is simply just an arithmetic mean of the individual coefficients estimated from each of the *M* analyses. We have

$$\bar{\theta }=\frac{\sum_{m=1}^{M}{\hat{\theta }}_{*m}}{M}.$$

Estimation of the standard error for each variable is a little more complicated. Let *V*_*W*_ be the within-imputation variance, which is the average of the variance of the estimated coefficient from each imputed dataset:

$$V_{W}=\frac{\sum_{m=1}^{M}\text{Var}\left({\hat{\theta }}_{*m}\right)}{M},$$

where $\text{Var}\left({\hat{\theta }}_{*m}\right)$ is the diagonal element of $\text{Var}\left(\hat{\theta }\right)$ calculated from Equation (4) using the imputed dataset. Let *V*_*B*_ be the between-imputation variance, which is calculated as

$$V_{B}=\frac{\sum_{m=1}^{M}{\left({\hat{\theta }}_{*m}-\bar{\theta }\right)}^{2}}{M-1}.$$

From *V*_*W*_ and *V*_*B*_, the variance of the pooled coefficients is calculated as

$$\text{Var}\left(\bar{\theta }\right)=V_{W}+V_{B}+\frac{V_{B}}{M}.$$

The above variance is statistically principled since *V*_*W*_ reflects the sampling variance and *V*_*B*_ reflects the extra variance due to the missing data.

Examples of metami() are provided as follows for the data from the Craft et al. (2003) meta-analysis in the previous section.


## prepare a dataset with missing values
Craft2003.mnar  <- Craft2003[, c(2, 4:10)]
Craft2003.mnar[sample(which(Craft2003$C4
 <  0), 6), "C4"]  <- NA

##  prepare input arguments for metami()
dat  <- Craft2003.mnar
n.name  <- "N"
ef.name  <- c("C1", "C2", "C3", "C4",
 "C5", "C6")


The number of imputations is specified through the argument M. The argument vcov controls the function to be used for computing the variance-covariance matrices for the effect sizes, whose options are vcov="r.vcov" for correlation coefficients and vcov="mix.vcov" for all the other types of effect sizes. For a random effect model, we can specify the argument func as "mixmeta", which allows the function mixmeta() from the package mixmeta to be used for MMA. For the argument func = "mixmeta", we have to specify formula and method for mixmeta().


library(mixmeta)
o2  <- metami(dat, M = 20, vcov = "r.vcov",
              n.name, ef.name,
              formula = as.formula(cbind
(C1, C2, C3, C4, C5, C6)~1),
              func = "mixmeta",
              method = "reml")


We could also use func = "meta" in the above code which adopts the function meta() from the metaSEM package, for which it is unnecessary to specify arguments formula and method.

For meta-regression, we can specify the name of the predictors in the argument x.name:


library(metaSEM)
o3  <- metami(dat, M = 20, vcov = "r.vcov",
              n.name, ef.name, x.name =
"p_male",
              func = "meta")


If we specify func = "mixmeta" in the above code, we also have to add p_male in the argument formula.

### 4.2. A simulation study for Craft et al.'s meta-analysis

The metavcov package provides several solutions for handling missing data. To compare these methods and find the influence of *M* in the MI method, a simulation study is conducted using the settings in the previous section. There are three missing data mechanisms, including missing completely at random (MCAR), missing at random (MAR), and missing not at random (MNAR). MCAR refers to the situation that neither the variables in the dataset nor the unobserved values of the variable itself predict whether a value will be missing; MAR refers to the circumstance that other variables (but not the variable with missing values itself) in the dataset can predict the missingness of a given variable; a variable is said to be MNAR if the value of the unobserved variable itself predicts missingness (Allison, 2001; Schafer and Graham, 2002; Graham, 2009; Mavridis and Salanti, 2013; Little and Rubin, 2019).

The code in the previous section simulated a missing data pattern of MNAR in C4, where only negative values were possibly missing. The MNAR scenario is the most challenging of the three. To check the performance of different methods, this procedure was replicated 100 times (*B* = 100). For the MI method, the number of imputations *M* was varied as 10, 20, 50, and 100. In addition to MI, methods of omitting the missing values (omission with mean imputed covariances) and single imputation with sample-size-weighted means (mean imputation) were also included. Recall that from equation (2), missingness in C4 could cause problems for the calculation of covariances between two other correlation coefficients, which makes an MMA impossible. Therefore, sample-size-weighted mean is used for imputing missing values in C4 for calculating covariances, which is achieved by specifying method = "average" in r.vcov().

Bias and mean squared error (MSE) were used to evaluate the methods for which the true parameter, denoted by θ^RE^, was defined as the estimated coefficient from the complete dataset using the function mixmeta() from the mixmeta package with its argument method = "reml". Let ${\bar{\theta }}_{b}$ be the estimated parameter using the imputed dataset from realization *b*. The bias and MSE were estimated by

$$\hat{\text{Bias}}\left(\theta^{\text{RE}}\right)=\frac{\sum_{b=1}^{B}\left({\bar{\theta }}_{b}-\theta^{\text{RE}}\right)}{B}\text{ and }\hat{\text{MSE}}\left(\theta^{\text{RE}}\right)=\frac{\sum_{b=1}^{B}{\left({\bar{\theta }}_{b}-\theta^{\text{RE}}\right)}^{2}}{B}.$$

In this dataset, we have *N* = 18 studies and the missing percentage in C4 is 33%. The effect sizes were transformed into Fisher's *z* scores. The R code for this simulation can be found in the supplementary material.

**Figure 6:**
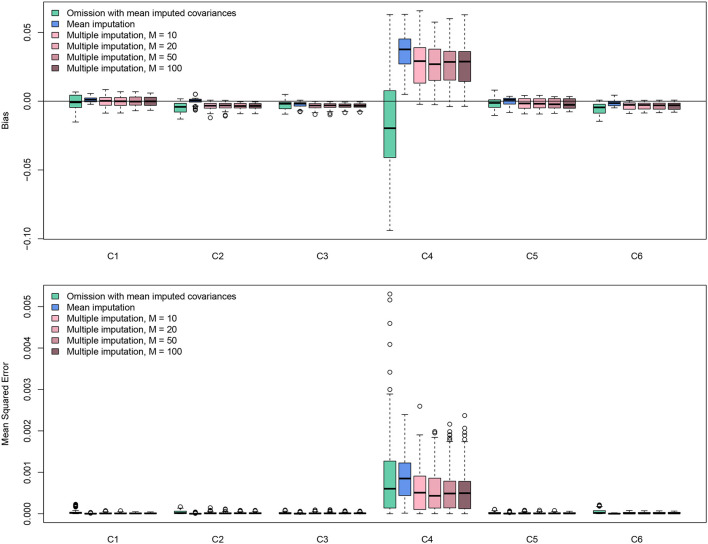
Bias and MSE results from simulation experiments using the data from the Craft et al. (2003) meta-analysis. Missing values in C4 were simulated in an MNAR pattern with 33% missing data; there is no missing value in C1, C2, C3, C5, and C6. The multiple imputation method conducted by the function metami from metavcov works better than other methods and *M* = 20 seems sufficient for this specific scenario.

The simulation results are displayed in Figure 6. The results were based on Fisher's *z* scores. All methods worked well since the values of bias were all roughly smaller than 0.002. For bias, the method of omission provided a smaller bias, but the results were highly variable. Mean imputation and MI methods gave more consistent results. Because smaller values were more likely to be missing, imputation methods tended to impute larger values based on observed data, generating positive bias. The mean imputation method had a higher bias, which caused higher values of MSE. The results showed that MI methods perform the best. Interestingly, the number of imputations *M* does not affect the result much. It seems that *M* = 20 is sufficient. Although missingness in C4 could influence the estimation of other effect sizes in terms of both bias and MSE, such influences are on a small scale. Overall, the missing value solutions from metavcov seem promising. Note that this conclusion is very specific to this dataset in this particular missingness pattern. The purpose of this section is to provide code (see supplemental data) for the users to conduct simulations for their own data to get some ideas of parameter settings and perhaps gain some confidence.

## 5. Summary and future work

The metavcov package provides useful tools for conducting MMA with examples in R under a generalizable, statistically principled analytical framework. It is very flexible in accommodating functions for different effect sizes and functions for different coefficient estimation methods. Compared with its earlier versions, functions have more consistent output values: all the model preparation functions, such as r.vcov and mix.vcov, store the outputs in ef, list.vcov, and matrix.vcov. It is very practical with functions for data visualization and handling missing values. As well as being statistically principled, it is helpful in practice that once the model has been specified, MI can be conducted automatically. In addition to end-users, developers can easily extend this package to other existing state-of-the-art trust models (Hedges et al., 2010; Chen et al., 2015, 2017; Tipton, 2015; Pustejovsky and Tipton, 2018).

The MI method was examined in an MNAR scenario from a simulation study. The MNAR scenario is very realistic for meta-analysis, which is also known as publication bias. Since published articles tend to show significant findings or be in favor of positive results, it is possible that imputing the missing effect sizes by zero could balance the findings and outperforms the MI method. The current version integrates the mice package for MI. Other packages for modeling missing data such as Amelia (Honaker et al., 2011) and mi (Su et al., 2011) may also be of users' choice for future work. Different estimation methods for random effect models, such as the method of moments or Bayesian approaches (Wei and Higgins, 2013a), should be compared as well for simulation studies. However, due to space limitations, they are not demonstrated in this article. From a theoretical perspective, no work has been done to calculate the covariances between correlation coefficients and other types of effect sizes, such as log odds ratio, which is also one of our future goals.

## Data availability statement

The original contributions presented in the study are included in the article/supplementary material, further inquiries can be directed to the corresponding author.

## Author contributions

The author confirms being the sole contributor of this work and has approved it for publication.

## References

[B1] Ahn S. Lu M. Lefevor G. T. Fedewa A. L. Celimli S. (2015). “Application of meta-analysis in sport and exercise science,” in An Introduction to Intermediate and Advanced Statistical Analyses for Sport and Exercise Scientists, eds. Ntoumanis N. Myers N. D. (New York: John Wiley Sons) 233–253.

[B2] Allison P. D. (2001). Missing Data. London: Sage publications. 10.4135/9781412985079

[B3] Aloe A. M. Amo L. C. Shanahan M. E. (2014a). Classroom management self-efficacy and burnout: A multivariate meta-analysis. Educ. Psychol. Rev. 26, 101–126. 10.1007/s10648-013-9244-0

[B4] Aloe A. M. Shisler S. M. Norris B. D. Nickerson A. B. Rinker T. W. (2014b). A multivariate meta-analysis of student misbehavior and teacher burnout. Educ. Psychol. Rev. 12, 30–44. 10.1016/j.edurev.2014.05.003

[B5] Barnard J. Rubin D. (1999). Miscellanea. Small-sample degrees of freedom with multiple imputation. Biometrika 86, 948–955. 10.1093/biomet/86.4.948

[B6] Becker B. J. (2000). “Multivariate meta-analysis,” in Handbook of Applied Multivariate Statistics and Mathematical Modeling, 499–525. 10.1016/B978-012691360-6/50018-5

[B7] Becker B. J. (2009). “Model-based meta-analysis,” in The Handbook of Research Synthesis and Meta-Analysis, eds. Cooper H. Hedges L. V. Valentine J. C. (Russell: Sage Foundation) 377–395.

[B8] Berkey C. Anderson J. Hoaglin D. (1996). Multiple-outcome meta-analysis of clinical trials. Stat. Med. 15, 537–557. 10.1002/(SICI)1097-0258(19960315)15:5<537::AID-SIM176>3.0.CO;2-S 8668877

[B9] Borenstein M. Hedges L. V. Higgins J. P. Rothstein H. R. (2021). Introduction to Meta-Analysis. New York: John Wiley &Sons. 10.1002/9781119558378

[B10] Boyles A. L. Harris S. F. Rooney A. A. Thayer K. A. (2011). Forest plot viewer: a new graphing tool. Epidemiology 22, 746–747. 10.1097/EDE.0b013e318225ba48 21811115

[B11] Chen H. Manning A. K. Dupuis J. (2012). A method of moments estimator for random effect multivariate meta-analysis. Biometrics 68, 1278–1284. 10.1111/j.1541-0420.2012.01761.x 22551393 PMC4030295

[B12] Chen Y. Hong C. Riley R. D. (2015). An alternative pseudolikelihood method for multivariate random-effects meta-analysis. Stat. Med. 34, 361–380. 10.1002/sim.6350 25363629 PMC4305202

[B13] Chen Y. Liu Y. Chu H. Ting Lee M.-L. Schmid C. H. (2017). A simple and robust method for multivariate meta-analysis of diagnostic test accuracy. Stat. Med. 36, 105–121. 10.1002/sim.7093 27580758 PMC6143393

[B14] Cheung M. (2021). Meta-Analysis using Structural Equation Modeling. R package version 1.2.5.1. 10.1093/acrefore/9780190224851.013.225

[B15] Cheung M. W.-L. (2008). A model for integrating fixed-, random-, and mixed-effects meta-analyses into structural equation modeling. Psychol. Method. 13, 182. 10.1037/a0013163 18778151

[B16] Cheung M. W.-L. (2009). Constructing approximate confidence intervals for parameters with structural equation models. Struct. Equat. Model. 16, 267–294. 10.1080/10705510902751291

[B17] Cheung M. W.-L. (2013). Multivariate meta-analysis as structural equation models. Struct. Equat. Model. 20, 429–454. 10.1080/10705511.2013.797827

[B18] Cheung M. W.-L. (2015). Meta-Analysis: A Structural Equation Modeling Approach. New York: John Wiley &Sons. 10.1002/9781118957813

[B19] Cichonska A. Rousu J. Marttinen P. Kangas A. J. Soininen P. Lehtimäki T.. (2016). metacca: summary statistics-based multivariate meta-analysis of genome-wide association studies using canonical correlation analysis. Bioinformatics 32, 1981–1989. 10.1093/bioinformatics/btw052 27153689 PMC4920109

[B20] Cooper H. Hedges L. V. Valentine J. C. (2009). The Handbook of Research Synthesis and Meta-Analysis. Russell Sage Foundation.

[B21] Craft L. L. Magyar T. M. Becker B. J. Feltz D. L. (2003). The relationship between the competitive state anxiety inventory-2 and sport performance: A meta-analysis. J. Sport Exer. Psychol. 25, 44–65. 10.1123/jsep.25.1.4412846532

[B22] Czernichow S. Kengne A.-P. Stamatakis E. Hamer M. Batty G. D. (2011). Body mass index, waist circumference and waist-hip ratio: which is the better discriminator of cardiovascular disease mortality risk? evidence from an individual-participant meta-analysis of 82 864 participants from nine cohort studies. Obes. Rev. 12, 680–687. 10.1111/j.1467-789X.2011.00879.x 21521449 PMC4170776

[B23] de Hollander E. L. Bemelmans W. J. Boshuizen H. C. Friedrich N. Wallaschofski H. Guallar-Castillón P.. (2012). The association between waist circumference and risk of mortality considering body mass index in 65-to 74-year-olds: a meta-analysis of 29 cohorts involving more than 58 000 elderly persons. Int. J. Epidemiol. 41, 805–817. 10.1093/ije/dys008 22467292 PMC4492417

[B24] Gasparrini A. (2019). Multivariate and Univariate Meta-Analysis and Meta-Regression. R package version 1.0.3.

[B25] Gasparrini A. Armstrong B. Kenward M. G. (2012). Multivariate meta-analysis for non-linear and other multi-parameter associations. Stat. Med. 31, 3821–3839. 10.1002/sim.5471 22807043 PMC3546395

[B26] Geeganage C. Bath P. M. (2010). Vasoactive drugs for acute stroke. Cochr. Datab. System. Rev. 2010, CD002839. 10.1002/14651858.CD002839.pub2 20614431 PMC7120409

[B27] Graham J. W. (2009). Missing data analysis: Making it work in the real world. Ann. Rev. Psychol. 60, 549–576. 10.1146/annurev.psych.58.110405.085530 18652544

[B28] Harville D. A. (1977). Maximum likelihood approaches to variance component estimation and to related problems. J. Am. Stat. Associ. 72, 320–338. 10.1080/01621459.1977.10480998

[B29] Hedges L. V. (1981). Distribution theory for glass's estimator of effect size and related estimators. J. Educ. Stat. 6, 107–128. 10.3102/10769986006002107

[B30] Hedges L. V. Tipton E. Johnson M. C. (2010). Robust variance estimation in meta-regression with dependent effect size estimates. Res. Synth. Methods 1, 39–65. 10.1002/jrsm.5 26056092

[B31] Higgins J. P. Thompson S. G. (2002). Quantifying heterogeneity in a meta-analysis. Stat. Med. 21, 1539–1558. 10.1002/sim.1186 12111919

[B32] Higgins J. P. Thompson S. G. Deeks J. J. Altman D. G. (2003). Measuring inconsistency in meta-analyses. BMJ 327, 557–560. 10.1136/bmj.327.7414.557 12958120 PMC192859

[B33] Higgins J. P. White I. R. Wood A. M. (2008). Imputation methods for missing outcome data in meta-analysis of clinical trials. Clin. Trials 5, 225–239. 10.1177/1740774508091600 18559412 PMC2602608

[B34] Honaker J. King G. Blackwell M. (2011). Amelia II: A program for missing data. J. Statist. Softw. 45, 1–47. 10.18637/jss.v045.i07

[B35] Hong C. Duan R. Zeng L. Hubbard R. A. Lumley T. Riley R. D.. (2020). The galaxy plot: a new visualization tool for bivariate meta-analysis studies. Am. J. Epidemiol. 189, 861–869. 10.1093/aje/kwz286 31942603 PMC7438574

[B36] Insua J. T. Sacks H. S. Lau T.-S. Lau J. Reitman D. Pagano D.. (1994). Drug treatment of hypertension in the elderly: a meta-analysis. Ann. Internal Med. 121, 355–362. 10.7326/0003-4819-121-5-199409010-00008 7726892

[B37] Jackson D. Riley R. White I. R. (2011). Multivariate meta-analysis: potential and promise. Stat. Med. 30, 2481–2498. 10.1002/sim.4172 21268052 PMC3470931

[B38] Jackson D. White I. R. Riley R. D. (2013). A matrix-based method of moments for fitting the multivariate random effects model for meta-analysis and meta-regression. Biometr. J. 55, 231–245. 10.1002/bimj.201200152 23401213 PMC3806037

[B39] Little R. J. Rubin D. B. (2019). Statistical Analysis With Missing Data. New York: John Wiley &Sons. 10.1002/9781119482260

[B40] Liu Q. Cook N. R. Bergström A. Hsieh C.-C. (2009). A two-stage hierarchical regression model for meta-analysis of epidemiologic nonlinear dose-response data. Comput. Stat. Data Analy. 53, 4157–4167. 10.1016/j.csda.2009.05.001

[B41] Lu M. (2017). Variance-Covariance Matrix for Multivariate Meta-Analysis. R package version 1.0.1.

[B42] Luo S. Chen Y. Su X. Chu H. (2014). mmeta: an r package for multivariate meta-analysis. J. Statist. Softw. 56, 1–26. 10.18637/jss.v056.i11 24904241 PMC4043353

[B43] Mavridis D. Salanti G. (2013). A practical introduction to multivariate meta-analysis. Stat. Methods Med. Res. 22, 133–158. 10.1177/0962280211432219 22275379

[B44] McShane B. B. Böckenholt U. (2022). Multilevel multivariate meta-analysis made easy: An introduction to mlmvmeta. Behav. Res. Methods. 17, 1–20. 10.3758/s13428-022-01892-7 35915358

[B45] Michael D. (2021). CRAN Task View: Meta-Analysis. London, United Kingdom: King's College London.

[B46] Nam I.-S. Mengersen K. Garthwaite P. (2003). Multivariate meta-analysis. Stat. Med. 22, 2309–2333. 10.1002/sim.1410 12854095

[B47] Nikoloulopoulos A. K. (2020). A multinomial quadrivariate d-vine copula mixed model for meta-analysis of diagnostic studies in the presence of non-evaluable subjects. Stat. Methods Med. Res. 29, 2988–3005. 10.1177/0962280220913898 32323626 PMC7682507

[B48] Olkin I. (1976). Asymptotic distribution of functions of a correlation matrix. J. Multiv. Analy. 11, 235–251.

[B49] Olkin I. Gleser L. (2009). “Stochastically dependent effect sizes,” in The Handbook of Research Synthesis and Meta-Analysis 357–376.

[B50] Pustejovsky J. E. Tipton E. (2018). Small-sample methods for cluster-robust variance estimation and hypothesis testing in fixed effects models. J. Busi. Econ. Stat. 36, 672–683. 10.1080/07350015.2016.1247004

[B51] R Core Team (2016). R: A Language and Environment for Statistical Computing. Vienna, Austria: R Foundation for Statistical Computing. ISBN 3-900051-07-0.

[B52] Riley R. D. (2009). Multivariate meta-analysis: the effect of ignoring within-study correlation. J. R. Stat. Soc. 172, 789–811. 10.1111/j.1467-985X.2008.00593.x

[B53] Rubin D. B. (1976). Inference and missing data. Biometrika 63, 581–592. 10.1093/biomet/63.3.581

[B54] Rubin D. B. (1987). Multiple Imputation for Nonresponse in Surveys. New York: John Wiley &Sons. 10.1002/9780470316696

[B55] Rücker G. Schwarzer G. (2021). Beyond the forest plot: The drapery plot. Res. Synth. Methods 12, 13–19. 10.1002/jrsm.1410 32336044

[B56] Schafer J. L. Graham J. W. (2002). Missing data: our view of the state of the art. Psychol. Method. 7, 147. 10.1037/1082-989X.7.2.147 12090408

[B57] Schwarzer G. (2007). Meta: An r package for meta-analysis. R NEWS 7, 40–45.

[B58] Sebri V. Durosini I. Triberti S. Pravettoni G. (2021). The efficacy of psychological intervention on body image in breast cancer patients and survivors: A systematic-review and meta-analysis. Front. Psychol. 12, 407. 10.3389/fpsyg.2021.611954 33732184 PMC7957010

[B59] Sedgwick P. (2015). How to read a forest plot in a meta-analysis. BMJ 351, h4028. 10.1136/bmj.h4028 26208517

[B60] Sera F. Armstrong B. Blangiardo M. Gasparrini A. (2019). An extended mixed-effects framework for meta-analysis. Stat. Med. 38, 5429–5444. 10.1002/sim.8362 31647135

[B61] Su Y.-S. Gelman A. Hill J. Yajima M. (2011). Multiple imputation with diagnostics (mi) in R: opening windows into the black box. J. Stat. Soft. 45, 1?-31. 10.18637/jss.v045.i02

[B62] Sutton A. J. Abrams K. R. Jones D. R. Jones D. R. Sheldon T. A. Song F. (2000). Methods for Meta-Analysis in Medical Research. Chichester: Wiley.

[B63] Thompson S. G. Smith T. C. Sharp S. J. (1997). Investigating underlying risk as a source of heterogeneity in meta-analysis. Stat. Med. 16, 2741–2758. 10.1002/(SICI)1097-0258(19971215)16:23<2741::AID-SIM703>3.0.CO;2-0 9421873

[B64] Tipton E. (2015). Small sample adjustments for robust variance estimation with meta-regression. Psychol. Method. 20, 375. 10.1037/met0000011 24773356

[B65] Torloni M. Betran A. Horta B. Nakamura M. Atallah A. Moron A.. (2009). Prepregnancy bmi and the risk of gestational diabetes: a systematic review of the literature with meta-analysis. Obesity Rev. 10, 194–203. 10.1111/j.1467-789X.2008.00541.x 19055539

[B66] Van Buuren S. Groothuis-Oudshoorn K. (2011). Mice: Multivariate imputation by chained equations in R. J. Statist. Softw. 45, 1–67. 10.18637/jss.v045.i03

[B67] Van Houwelingen H. C. Arends L. R. Stijnen T. (2002). Advanced methods in meta-analysis: multivariate approach and meta-regression. Stat. Med. 21, 589–624. 10.1002/sim.1040 11836738

[B68] Viechtbauer W. (2010). Conducting meta-analyses in r with the metafor package. J. Statist. Softw. 36, 1–48. 10.18637/jss.v036.i03

[B69] Viechtbauer W. (2021). Multiple Imputation With the Mice and metafor packages.33961037

[B70] Watters E. R. Aloe A. M. Wojciak A. S. (2021). Examining the associations between childhood trauma, resilience, and depression: a multivariate meta-analysis. Trauma Viol. Abuse 24, 231–244. 10.1177/15248380211029397 34313169

[B71] Wei Y. Higgins J. P. (2013a). Bayesian multivariate meta-analysis with multiple outcomes. Stat. Med. 32, 2911–2934. 10.1002/sim.5745 23386217

[B72] Wei Y. Higgins J. P. (2013b). Estimating within-study covariances in multivariate meta-analysis with multiple outcomes. Stat. Med. 32, 1191–1205. 10.1002/sim.5679 23208849 PMC3618374

[B73] Winter J. E. MacInnis R. J. Wattanapenpaiboon N. Nowson C. A. (2014). Bmi and all-cause mortality in older adults: a meta-analysis. Am. J. Clin. Nutr. 99, 875–890. 10.3945/ajcn.113.068122 24452240

[B74] Yuan Y. Little R. J. (2009). Meta-analysis of studies with missing data. Biometrics 65, 487–496. 10.1111/j.1541-0420.2008.01068.x 18565168

